# A Quantum Mechanism Study of the C-C Bond Cleavage to Predict the Bio-Catalytic Polyethylene Degradation

**DOI:** 10.3389/fmicb.2019.00489

**Published:** 2019-03-12

**Authors:** Junyu Xu, Ziheng Cui, Kaili Nie, Hao Cao, Min Jiang, Haijun Xu, Tianwei Tan, Luo Liu

**Affiliations:** ^1^Beijing Key Laboratory of Bioprocess, Beijing University of Chemical Technology, Beijing, China; ^2^Laboratory of Biomanufacturing and Food Engineering, Institute of Food Science and Technology, Chinese Academy of Agricultural Sciences, Beijing, China; ^3^State Key Laboratory of Materials-Oriented Chemical Engineering, College of Biotechnology and Pharmaceutical Engineering, Nanjing University of Technology, Nanjing, China

**Keywords:** polyethylene, oxidation, carbon-carbon bond cleavage, quantum mechanism, bond dissociation energy (BDE)

## Abstract

The growing amount of plastic solid waste (PSW) is a global concern. Despite increasing efforts to reduce the residual amounts of PSW to be disposed off through segregated collection and recycling, a considerable amount of PSW is still landfilled and the extent of PSW ocean pollution has become a worldwide issue. Particularly, polyethylene (PE) and polystyrene (PS) are considered as notably recalcitrant to biodegradation due to the carbon-carbon backbone that is highly resistant to enzymatic degradation via oxidative reactions. The present research investigated the catalytic mechanism of P450 monooxygenases by quantum mechanics to determine the bio-catalytic degradation of PE or PS. The findings indicated that the oxygenase-induced free radical transition caused the carbon-carbon backbone cleavage of aliphatic compounds. This work provides a fundamental knowledge of the biodegradation process of PE or PS at the atomic level and facilitates predicting the pathway of plastics’ biodegradation by microbial enzymes.

## Introduction

Plastics are widely used in industrial and household applications because of their low weight, durability and low production cost (Andrady, 2015). However, the growing amount of plastic solid waste (PSW) is a global concern. The widespread use of plastics, the lack of waste management and casual social behavior, however, pose a major threat to the environment (Leja and Lewandowicz, 2010). Despite increasing efforts to reduce the residual amounts of PSW to be disposed off through segregated collection and recycling, a considerable amount of PSW is still landfilled and the extent of PSW ocean pollution has become a worldwide issue (Baeyens et al., 2010; Brems et al., 2012).

Considering their abundance of plastics in the environment, biodegradation of plastics could be the most effective way. Decades ago, several biodegradable aliphatic polyesters such as PLA and PHB, were produced to replace petrochemical plastics (Tokiwa et al., 2009). However, the most commonly used plastics are still synthetic polymers obtained from petrochemical hydrocarbons and derivatives (Geyer et al., 2017). Polyethylene (PE) and polystyrene (PS) are amongst the most important mass-produced plastics and largely manufactured into short-life products including packaging materials for food and disposable dishware (Plastics Europe, 2018). PE and PS are highly stable polymers and notably resistant to biodegradation (Ho et al., 2017). The carbon-carbon backbone in PE and PS is highly resistant to enzymatic cleavage by oxidation-reduction (Goldman, 2010). Additionally, the high molecular weight and strong hydrophobic character hamper their biodegradation (Albertsson and Karlsson, 1993).

Recently, several microbes and microbial enzymes have been shown able to catalyze the degradation of various petrochemical plastics including PE and PS (Wei and Zimmermann, 2017). Shimpi et al. (2012) reported the biodegradation of modified PS by using a pure strain of *Pseudomonas aeruginosa*. Motta et al. (2009) used the *Curvularia* species to investigate the degradation of atactic PS. These results suggested that the biodegradation of PS material through using selected microbial strains might become a feasible solution for reducing the huge amount of waste and disposed plastics.

Sivan et al. isolated the actinomycete *Rhodococcus ruber* (C208) to degrade PE and PS (Mor and Sivan, 2008; Santo et al., 2013), and demonstrated that laccase, a copper-binding enzyme, played a crucial role in the oxidation and degradation of PE by *R. ruber* (Santo et al., 2013). In addition to laccase, several oxidoreductases were shown to be involved in the biodegradation of PE and PS, such as the AlkB family hydroxylases and hydroquinone peroxidase (Nakamiya et al., 1997; Jeon and Kim, 2015).

The catalytic mechanism of oxidoreductases with respect to the cleavage of PE and PS still remains unexplained. The present work applied quantum mechanism calculations to unveil the bio-catalytic mechanism of PE and PS degradation by oxidoreductase, with the P450 monooxygenase catalyzed reaction being treated as a typical saturated carbon-carbon bone cleavage reaction (Matthews et al., 2017). This work attempts to provide fundamental insights into the possible biodegradation of plastics with a C-C backbone.

## Materials and Methods

### Computation Methods

In this work, geometry optimizations, relaxed scan and natural population analysis (NPA) charges were calculated by the Gaussian 09 software package (Frisch et al., 2013) at the B3LYP/6-31+G(d,p) theoretical level. The frequency of structures was also calculated at same level to ensure that the stable structures have no imaginary frequency, and only one imaginary frequency for the transition state. Fuzzy bond orders, spin density and spin population analysis were calculated by Multiwfn (Lu and Chen, 2012). Relaxed force constants were calculated by Compliance (Brandhorst and Grunenberg, 2008; Brandhorst and Grunenberg, 2010).

### Analysis of Three Bond Strength Descriptors

The bond dissociation energy (BDE), widely used in the literature as a kind of bond strength descriptor can be defined as the standard enthalpy (H) change when a bond is cleaved by homolysis to produce two fragments. In some cases, it is however better to calculate the standard enthalpy change when a bond is cleaved by heterolysis. These calculations of Bond Dissociation Energy (BDE, at 298K) are shown in the following equation:

$$BDE(298K)=H(RH_{2}•)+H(•COOH)-H(RCH_{2}-COOH)$$

However, the numerical value of BDE, as an intrinsic strength of a particular bond, depends on the stable molecule and the stability of the fragments, such as electronic ground state, minimum conformation, etc. (Grunenberg, 2017). The bond order is a quantitative description of chemical bonds and has been widely used by chemists to understand the nature of molecular electronic structure and predict the molecular reactivity, aromaticity, and stability (Lu and Chen, 2013). The fuzzy bond order exhibits very little basis set sensitivity and will not be deteriorated by using diffuse basis functions (Mayer and Salvador, 2004). For the same type of bonds, the fuzzy bond order positively correlates to bond strength.

Force constants are widely used as an intuitive bond strength descriptor. However, the numerical values of rigid force constants depend on the choice of coordinate systems. In order to overcome this disadvantage and achieve a higher precision, the compliance matrix, which is a second-order tensor containing non-zero coupling elements (Grunenberg, 2017), was adopted in this work to describe the bond strength.

## Results and Discussion

### Model Structures and Charge Distribution Analysis

**FIGURE 1:**
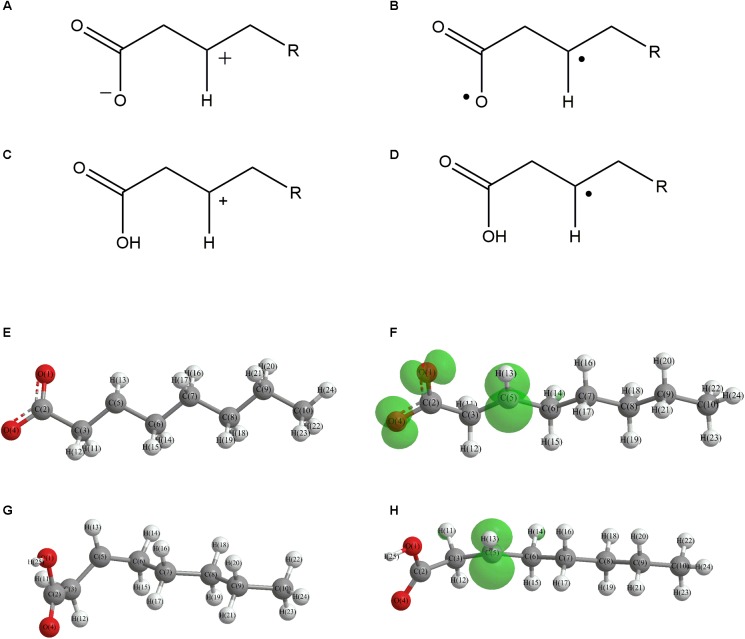
Molecular structure: **(A)** Ionic structure in alkaline condition; **(B)** Free radical structure in alkaline condition; **(C)** Ionic structure in acidic condition; **(D)** Free radical structure in acidic condition. **(E–H)** are corresponding spin density for molecules **(A–D)**, which was marked with green. The value of the iso-surface is 0.01. The carbon atoms, C2 is the carboxylic carbon atom, C3 is the alpha carbon.

In this work, the structures of Figure 1 were investigated to determine the cleavage of the C-C bond close to the carboxyl group under both acidic or alkaline conditions. Since the four molecules are very similar, spin density and NPA charges were calculated to ensure that these structures are reliable. The bond length of the C2-C3 bonds (C3 is the alpha carbon, C2 is the carboxylic carbon atom) in the molecules and the NPA charges of certain atoms are given in Table 1. It is specifically mentioned here that for the structure shown in Figure 1A,E, a stable configuration is not obtained after a plenty of structural optimizations. The C2-C3 bond cleavage occurred in every optimization attempt with molecule Figure 1E as the initial structure. Therefore, structures with optimization are adopted as the basis for this part of the study.

**Table 1:** Bond lengths of C2-C3 bonds and certain atomic NPA charges.

molecule	bond lengths of C2-C3 bonds/ Å	NPA charges of C5/a.u.	NPA charges of O1/a.u.	NPA charges of O4/a.u.
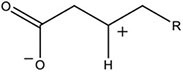	1.569	0.173	-0.625	-0.579
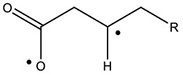	1.502	-0.128	-0.473	0.388
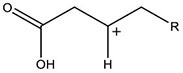	1.551	0.230	-0.506	-0.723
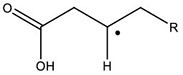	1.515	-0.124	-0.735	-0.604


The results of the spin density in Figure 1 show that there are no unpaired electrons in the ionic structures, while some unpaired electrons exist in the free radical structures mainly in O1, O4, and C5. As shown in Table 1, NPA charges of the C5s in ionic structures for both an alkaline and an acidic environment are positive, while the NPA charges are negative for the two other structures. Compared to the NPA charges of oxygen atoms of the ionic structure, there are more negative charges in the oxygen atom O1 in ionic structure in alkaline environment. Therefore, these structures are appropriate for further calculations.

### Effect of the Structure on C-C Bond Cleavage

#### The Ionic Structure Under Alkaline Condition

For structure (A) of Figure 1, no stable corresponding structure was obtained after several rounds of optimization. Therefore, an optimized free fatty acid was used as template, a hydride ion at C_β_ was removed to obtain the approximate structure for further calculation. The approximate structure is shown in Figure 1E.

**FIGURE 2:**
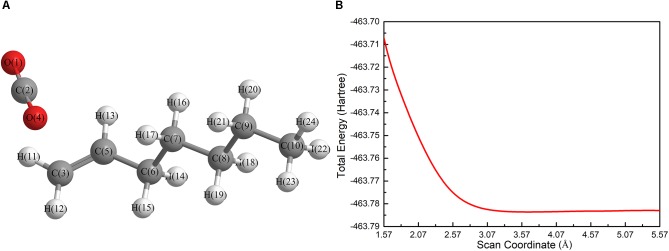
**(A)** Ionic structure in alkaline condition after optimization; **(B)** the result of relaxed scan along the distance of the C2-C3 bond based upon the unstable structure as shown in Figure 1E. The unit in **(B)** is Hartree, 1 Ha = 2625.499638(65) kJ/mol.

Firstly, a geometry optimization was performed for this structure, and the result is shown in Figure 2A. It is obvious that the molecule is cleaved into a linear olefin and CO_2_ which are obtained from the cleavage of the fatty acid carboxylate. Then, a potential energy surface relaxed scan was performed along the distance of the C2-C3 bond in the molecule as shown in Figure 1E, to describe the energy of this system. The result of the scan is shown in Figure 2B, demonstrating that the energy decreases quickly as the distance increases initially, which indicates that the C2-C3 bond in the molecule as shown in Figure 1A is very unstable.

**FIGURE 3:**
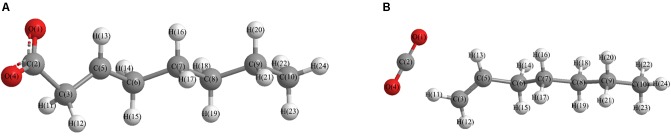
**(A)** Optimized transition state structure, based upon the unstable structure as shown in Figure 1E; **(B)** One stable structure connects the transition state structure.

For a more thorough study, the transition state which is shown in Figure 3A, was also calculated based upon the unstable structure as shown in Figure 1E. The vibration direction of the imaginary frequency is mainly along the direction of the C2-C3 bond stretching. A stable structure with a minimum in potential energy surface and connecting the transition state structure was calculated as shown in Figure 3B. The stable structure consists of a linear olefin and CO_2_ as the products derived from the cleavage of fatty acids. The energy of the molecule, as shown in Figure 1E is 110.7 kJ/mol higher than the energy of the transition state, which confirms the high instability of the molecule as shown in Figure 1E. Through the above calculations, it is clear that the ionic structure in an alkaline environment is close to the transition state structure in its potential energy surface, which indicates the instability of the C2-C3 bond.

#### The Free Radical Structure Under Alkaline Condition

**FIGURE 4:**
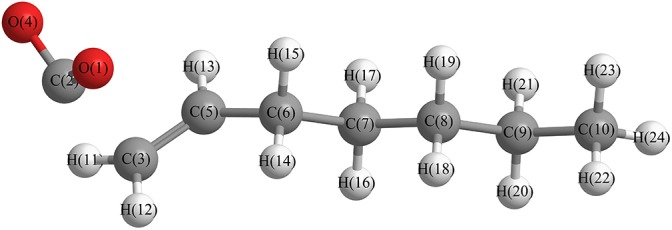
Optimized transition structure, based upon the free radical structure in alkaline condition.

Based upon the free radical structure in an alkaline environment as shown in Figure 1F, a transition state optimization was carried out. The change of spin multiplicity caused by the cleavages of C2 and C3 did not consider here. The distance of C2-C3 in this transition structure is 2.23 Å, which means that this molecule is divided into two parts. Table 2 and Figure 4 show the result of the spin population analysis which was carried out to determine the distribution of single electrons in the transition structure. This analysis shows that more than 80% (about two electrons) of single electrons are on the part of the free carboxyl group, indicating that a linear olefin was produced. Assessing the normal mode corresponding to the imaginary frequency, it was found that the composing displacements tend to lead in the directions of the structure derived from the cleavage of the fatty acid carboxylate. Therefore, the transition state structure connects the product consisting of a linear olefin and CO_2_. The energy of the transition state includes zero-point correction and is 109.8 kJ/mol relative to the molecule as shown in Figure 1F. Due to the high energy of the transition state, it is not easy to cleave the bond in this structure.

**Table 2:** Result of spin population analysis based on the transition structure shown in Figure 4.

Atomic space	Value	% of sum	% of sum abs
O1	0.64107952	32.054013	30.046278
C2	0.42337472	21.168761	19.842834
C3	-0.06553244	-3.276626	-3.071391
O4	0.64994558	32.497317	30.461815
C5	0.30392666	15.196351	14.244512


As can be seen from Table 3, when a hydrogen atom has been removed from C_β_, the changes of BDEs, relaxed force constants and the bond orders are very small. So the C2-C3 bond strengths in both structures are very similar and the lack of a hydrogen atom at the location of C_β_ has little influence on the C2-C3 bond strength.

**Table 3:** Bond orders, relaxed force constants and BDEs of the C2-C3 bonds in the selected molecules.

Selected molecules	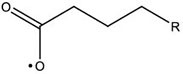	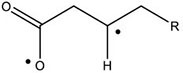	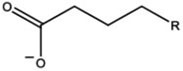
bond orders of C2-C3 bond	1.073	1.065	0.98
relaxed force constants of C2-C3 bond (mdyn/Å)	4.237	4.184	2.941
BDE (kJ/mol)	351.86	350.07	279.84


By comparing the third column with the fourth column in Table 3, it is obvious that the bond order, relaxed force and BDE of the C2-C3 bond in the free radical structure in an alkaline environment are much higher than the C2-C3 bond in a carboxylate anion, which indicates that the C2-C3 bond strength of the free radical structure in an alkaline environment is stronger. Because decarboxylation is less favorable at low temperatures and highly sensitive to conditions for carboxylic acids, decarboxylation of the molecule in Figure 1F is more difficult.

#### Ionic Structure Under Acidic Condition

If there is a linear olefin produced, the C2-C3 bond in this structure should be cleaved by heterolysis, and hence Table 4 gives the heterolytic bond dissociation energies of the C2-C3 bonds. By comparing the second and the fourth columns in Table 4, it can be seen that the values of the bond order, relaxed force constant, BDE and heterolytic bond dissociation energy of the C2-C3 bond become much smaller when a hydrogen anion is removed from C_β_ in a carboxylic acid, which indicates that the bond strength becomes much weaker. Comparing the fourth column in Table 4 to the fourth column in Table 3, it can be seen that the values of the bond orders, the relaxed force constants and the BDEs of the C2-C3 bonds are very close, so the C2-C3 bond strengths in the two structures are basically the same.

**Table 4:** Bond orders, relaxed force constants, BDEs and heterolytic bond dissociation energies of the C2-C3/O-H bonds in the structures of selected molecules.

Selected molecules	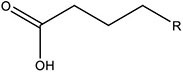		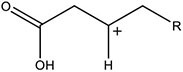	
Bonds	C2-C3	O-H	C2-C3	O-H
bond order of C2-C3/O-H bond	1.039	0.833	0.965	0.807
relaxed force constant of C2-C3/O-H bond(mdyn/Å)	4.115	7.813	2.915	7.692
BDE(kJ/mol)	363.32	–	269.58	–
heterolytic bond dissociation energy(kJ/mol)	1173.24	1437.5	216.08	748.33

**Table 5:** Bond orders, relaxed force constants, BDEs and heterolytic bond dissociation energies of the C2-C3/O-H bonds in the structures of selected molecules.

Selected molecules	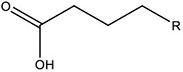		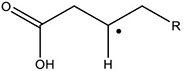	
Bonds	C2-C3	O-H	C2-C3	O-H
bond order of C2-C3/O-H bond	1.039	0.833	1.033	0.831
relaxed force constant of C2-C3/O-H bond(mdyn/Å)	4.115	7.813	3.968	7.813
BDE(kJ/mol)	363.32	–	361.13	–
heterolytic bond dissociation energy(kJ/mol)	–	1437.50	–	1422.34


As the absence of a hydrogen anion in a carboxylic acid may cause the O-H bond to become weaker, it was decided to investigate the O-H bond strength of the ionic structure in an acidic environment and determine whether it has an impact on the decarboxylation. As can been seen from Table 4, when a hydrogen anion is removed from C_β_ in a carboxylic acid, the values of the bond order and the relaxed force constant of the O-H bond decrease slightly and the heterolytic bond dissociation energies of the O-H bond become much smaller. The reason why the heterolytic bond dissociation energy varies significantly may be due to the major configuration changes from the segmentation optimization calculation (C-C bond cleavage), which affects the calculation of the heterolytic bond dissociation energy. In summary, the absence of a hydride ion does not significantly affect the strength of the O-H bond and therefore does not affect C-C bond cleavage.

#### Free Radical Structure Under Acidic Condition

Table 5 shows that the bond order, relaxed force constant, and BDE of C2-C3 bond are not significantly changed in the absence of a hydride atom at C_β_ position. Moreover, the bond order, relaxed force constant, and BDE of this structure are much larger than the corresponding value of molecule in the fourth column of Table 3. Therefore, the bond strength of C2-C3 bond in this structure is very high. Table 5 also shows that the bond order, relaxed force constant, heterolytic bond dissociation energy of O-H bond do not change significantly, in the absence of a hydride atom at C_β_ position, indicating that the bond strength of the O-H bond does not change significantly, and hence does not affect C-C bond cleavage.

In summary, in the ionic structure, the absence of a hydrogen anion at the C_β_ position significantly reduces the bond strength of C2-C3. Therefore, whether in an acidic environment or an alkaline environment, C-C bond cleavage is more likely to occur in the ionic structures. On the other hand, in an alkaline environment, the C2-C3 bond is more unstable because of the presence of a carboxylate anion that promotes the tendency of push electrons. Therefore, the ionic structure under alkaline conditions is most advantageous for the removal of carboxyl groups.

## Conclusion

The enzymatic cleavage of C-C bond in aliphatic compounds by quantum mechanism calculation was investigated. Under certain conditions, the enzyme could abstract a hydrogen anion from the aliphatic compounds, causing the absence of a hydride anion at the C_β_ position, which significantly reduces the bond strength of C2-C3 bond and finally results in the C-C bond cleavage. The results reveal that oxidase or oxygenase could be involved in the C-C bond cleavage in PE/PS, thus facilitating their biodegradation.

## Data Availability

All datasets generated for this study are included in the manuscript and/or the supplementary files.

## Author Contributions

TT, MJ, HX, and LL conceived and designed the experiments. JX and ZC carried out the experiments. JX, ZC, KN, and HC analyzed the data. All authors wrote the manuscript.

## Conflict of Interest Statement

The authors declare that the research was conducted in the absence of any commercial or financial relationships that could be construed as a potential conflict of interest.
